# Relation of Board of Health to the City and Medical Profession

**Published:** 1899-12

**Authors:** R. R. Kime

**Affiliations:** Atlanta, Ga.; President Tri-State Medical Society of Georgia, Alabama and Tennessee; Ex-President Atlanta Society of Medicine


### RELATION OF BOARD OF HEALTH TO THE CITY AND MEDICAL PROFESSION.*

By R. R. KIME, M.D., Atlanta, Ga.

President Tri-State Medical Society of Georgia, Alabama and Tennessee; Ex-President Atlanta Society of Medicine.

*To the Atlanta Society of Medicine:*

As chairman of your committee I desire to offer a few remarks as a plea for a closer relation and stronger unity between the Board of Health, the city and the medical profession of Atlanta.

The Board of Health is an organized body elected by the city council to look after the health and sanitary condition of the city, executing methods and means of preventing diseases and epidemics and controlling the same. Each member is selected because of his fitness and qualifications to discharge such duties regardless of politics and political favors. The physicians on such boards should be broad in their views, representative men in their profession, with a knowledge of sanitary science that will render them able to cope with the grave problems in the prevention of disease and epidemics.

It is one of the most important boards in the city government, as nothing will destroy a city and its prospects more rapidly than the ravages of disease and epidemics. The benefits of a health board cannot be measured in dollars and cents; but the lack of a competent one, well equipped, entails a tenfold loss of dollars and cents to a city and its citizens, which is not to be compared to the loss of useful men, the saddening of happy homes, the suffering and destruction of innocent ones. Nowhere in the whole realm of human economy does the adage "an ounce of prevention is worth a pound of cure" apply more aptly than to the money properly utilized by a competent board of health. If a board of health is not competent it is the fault of the council that created it.

It is shortsighted economy to create a board of health and then prevent its doing efficient work for lack of funds. A city is responsible for the protection of its citizens and is derelict of its

*Read before the Atlanta Medical Society, November, 1899.

duty when it fails to provide protection as far as possible from disease and epidemics.

I am told Atlanta has pointed with pride, heretofore, to her health record, but this year the standard is being lowered, and why? The most important reason is for the lack of funds to carry out efficiently the workings of the Board of Health.

I am informed but little garbage has been burned this year, that it is thrown out at different points around the city to decay, and the drainage from it mixed with the sewerage which waters scores of milk cows around the city, from which some of the dairies and citizens get their supply of milk. The decomposition of garbage, contamination of water and milk supply will produce other diseases as well as typhoid fever. I think I would be safe in saying hundreds of cows drink this contaminated water when herded around the city by boys and pastured on farms. Hence the necessity of strict sanitary regulations as to milk supply, not on account of its dilution with water, but its infection and adulteration, including vessels in which it is collected and delivered.

In passing we would suggest that the Board of Health should, as far as possible, prevent the sale of milk from tuberculous cattle; in fact the State should make such requirements.

As the city grows older and more populous disease will increase, epidemics become more common, unless sanitary measures are rigidly inforced. To accomplish this in a city the size of Atlanta it will not only be necessary to give the board of health our hearty support and supply it with funds, but also secure a properly equipped bacteriological and chemical laboratory. Such a laboratory is a necessity and should be provided by the city without delay. It would be of incalculable value and assistance in the diagnosis of contagious disases and in preventing epidemics.

The benefits to be derived from the culture and microscopical tests in diphtheria alone would almost justify its establishment; but when we consider its necessity in the investigation of the milk supply, water supply, contaminated wells, diagnosis of cases of consumption, its part in detecting deficient plumbing and sewerage, there is no reasonable doubt as to its utility.

If the city council fully realized the benefits to be derived from such a laboratory I do not see how they could as representatives of the interests of the city of Atlanta and her citizens, refuse to appropriate sufficient funds to equip and conduct such a laboratory.

He who refuses to appropriate such funds and give his encouragement and support to the board of health may soon have cause for regret in the visitation of a preventable disease in his own home.

Let us all protect the fair name of the city of Atlanta by maintaining her health standard, encourage, equip and sustain her health board in its efforts to prevent and control disease in our midst.

Much good has been accomplished in the past and greater good can be accomplished in the future if we will all unite in our support. The board deserves the thanks of the citizens of Atlanta, especially for the manner in which they have controlled the last two or three epidemics of smallpox in our city.

I regret to say the medical profession has not given the board that support it should. I am inclined to believe that lack of support is due to indifference and neglect rather than opposition.

The great majority of physicians are kind-hearted, generous and true to the trusts committed to their care, and ever ready to prevent as well as cure disease; ever ready to give of their knowledge and counsel for the good of the general public.

I know by personal experience when busy how prone we are to procrastinate and forget to make reports that are essential to the board of health if it keep accurate statistics. If the statistics are unreliable, inaccurate and deficient, it is the fault of the profession in not reporting promptly. If a physician fails to report a case of smallpox other people are exposed and an epidemic ensues. The same with measles, scarlet fever, diphtheria, etc. If he fails to report typhoid fever others may become infected, while the cause remains unknown and unremoved and a menace to the general public.

A prompt, accurate diagnosis with report to Board of Health is to be desired in all contagious diseases. We all know how difficult it is at times to make a positive diagnosis in scarlet fever, diphtheria, typhoid fever, etc. It is here a properly equipped laboratory, in charge of a medical health officer who is an expert microscopist and bacteriologist, is essential for the control and prevention of disease. One versed in sanitary science, with some practical experience in the diagnosis of contagious diseases. One competent to take a specimen from a case of diphtheria and demonstrate by culture test and microscopically the presence or absence of the Klebs-Lœffler bacillus as well as the streptococcus. One competent to demonstrate the Widal test or Diazo reaction in typhoid fever, confirming or rejecting suspected cases. One competent and equipped to investigate the water, milk, and food supply, analyzing the same chemically and microscopically investigating the sanitary condition, plumbing, etc., ferreting out the cause of disease so it can be removed before others are infected.

A laboratory and medical health officer for the study and prevention of typhoid fever for the city of Atlanta would be a good investment, and doubly so when we consider the question of the prevention of diphtheria, scarlet fever, smallpox, measles, and the host of septic infectious conditions produced by defective plumbing and sewerage, such infection even invading the lying-in chamber, infecting woman during the most trying ordeal of her life. The plumbing and sewerage should be under the supervision of a physician versed in sanitary science. Diphtheria, scarlatina, and typhoid fever are becoming too common in our city, and more active, stringent measures should be instituted to prevent their propagation and spreading. Consumption (tuberculosis) should also be investigated and measures instituted to prevent its spread. Every case should be reported, and the family instructed in methods of prevention. The increasing population, influx of people with lung trouble, the physical degeneration of the colored race, make this a problem of no little importance to the future welfare of Atlanta.

The medical health officer should be one selected on account of his fitnes and qualification to fill the place. His selection should not be controlled by any political faction, and removed as far from politics as possible. He should be appointed for a term of not less than three or five years so as to do the most efficient work. Such an officer should be recommended by the medical profession of the city, as they are better prepared to judge of the qualifications of an applicant to properly fill the office.

Now a few words as to the general public. The greater majority, especially of the more enlightened, would willingly cooperate with the Board of Health and health officer if they only understood their duty and the benefits to be derived from such cooperation. I believe it to be the duty of the Board of Health to furnish that instruction for the benefit of the city and preventing the spread of disease. A printed slip containing a few concise, plain, practical statements as to manner in which contagion is spread in each disease, the best methods of prevention with printed formula of the most efficient, economical disinfectant solutions, with manner of using same, should be sent each and every family by the Board of Health as soon as they receive notice of a contagious disease. Such slip should also contain all laws, rules and regulations of the city and Board of Health relative to the disease for which notice is sent. Such a course would enlighten the general public and go far toward securing their assistance and cooperation with the attending physician, lessening his labors, and ofttimes preventing the spread of contagion, thus saving the suffering and lives of many innocent ones.

In conclusion I would urge that we as a profession give the Board of Health that assistance and support it needs and deserves. The Board of Health cannot accomplish that which it should without the support of the profession and public. The profession is responsible for its apathy and indifference in this matter, and to a great extent moulds the public opinion, and is in a measure responsible for the increase of contagious diseases in the city of Atlanta. As citizens, members of a noble profession, and in the interest of suffering humanity, let us discharge our duty in sustaining, aiding and encouraging the Board of Health. It is one of the most important boards in municipal affairs, but if its workings are obstructed by the profession, its hands tied by the city council, disease and death will follow.

So far this year there have been reported 137 cases of scarlet fever, 22 of diphtheria, 23 of membranous croup, 259 of typhoid fever, 272 of measles, making a total of 705 cases of preventable diseases, beside those that have not been reported. Something must be done to check the progress of contagious diseases in Atlanta, or as we grow older we will become a hotbed of contagion.

The Board of Health can tell you the troubles they encounter, the superintendent, principals, and teachers can inform you of the difficulties they meet with in trying to prevent the spread of contagion, the lack of sympathy and support from the profession and general public. Harmony should exist all along the line, all should unite in their workings for the good of humanity, lessening suffering and building up of the health record of Atlanta.

